# Antibiotic resistance of *Escherichia coli* and *Salmonella* spp. strains isolated from healthy poultry farms in the districts of Abidjan and Agnibilékrou (Côte d’Ivoire)

**DOI:** 10.14202/vetworld.2021.1020-1027

**Published:** 2021-04-28

**Authors:** Moumouni A. Assoumy, André P. Bedekelabou, Assiongbon Teko-Agbo, Walter Ossebi, Komlan Akoda, Félix Nimbona, Stanislas H. Zeba, Anicet A. Zobo, Raoul C. T. Tiecoura, Vessaly Kallo, Komissiri Dagnogo, Rianatou Bada-Alambédji

**Affiliations:** 1 Pharmacy-toxicology service, Department of Public Health and Environment, Inter-State School of Veterinary Sciences and Medicine (EISMV), BP 5077 Dakar, Senegal; 2 Microbiology, Immunology and Infectious Pathology Service, Department of Public Health and Environment, EISMV of Dakar, Senegal; 3 Rural Economy and Management Service, Department of Biological Sciences and Animal Productions, EISMV of Dakar, Senegal; 4 Directorate of Veterinary Services, Abidjan, Côte d’Ivoire; 5 Animal Health and Veterinary Public Hygiene Improvement Project (PASA-HPV), Abidjan, Côte d’Ivoire

**Keywords:** antibiotic resistance, Côte d’Ivoire, *Escherichia coli*, poultry, *Salmonella* spp

## Abstract

**Background and Aim:**

Antimicrobial resistance (AMR) is a serious challenge to animal and human health worldwide. Therefore, this study aims to determine levels and patterns of AMR of *Escherichia coli* and *Salmonella* spp. strains isolated from poultry farms in Côte d’Ivoire.

**Materials and Methods:**

A cross-sectional study was conducted in two districts of Côte d’Ivoire with high poultry production: Abidjan and Agnibilékrou. A total of 231 fecal samples were collected in 124 poultry farms in both districts. Enterobacteria were isolated and tested for susceptibility to 14 antimicrobial agents using the disk-diffusion method.

**Results:**

A total of 212 *E. coli* and 36 *Salmonella* strains were isolated. In Abidjan, 139 collected samples generated 101 *E. coli* and 23 *Salmonella* strains, whereas in Agnibilékrou, 92 collected samples generated 111 *E. coli* and 13 *Salmonella* strains. Variable resistance levels were recorded for the antibiotics tested. The resistance prevalence of *E. coli* and *Salmonella*, respectively, was high: Doxycycline (98%/94%), sulfonamide (84%/86%), trimethoprim-sulfamethoxazole (80%/41%), and streptomycin (71%/52%). Average resistance rates were recorded for flumequine (38%/66%), ampicillin (49%/33%), amoxicillin (25%/44%), colistin (26%/2%), chloramphenicol (21%/2%), and gentamicin (4%/47%). The antibiotics least affected by resistance were cefuroxime (4%/5%), ceftriaxone (2%/0.00%), and nitrofurantoin (1%/0.00%).

**Conclusion:**

In this study, it was observed that resistance to important antibiotics is emerging in poultry production in Côte d’Ivoire. Policies promoting the rational use of antibiotics should be implemented to manage antibiotic resistance in animal production.

## Introduction

The presence of bacterial resistant and multi-resistant to antibiotics causes problems in breeding (therapeutic failures) and human health [1,2]. Antibiotic resistance from animal sources can negatively affect human health, either directly or indirectly [3]. The direct effects result from the resistance of bacteria in human infections caused by microorganisms transmitted from animals to humans (zoonosis), typically through food [4], whereas indirect effects occur when resistance genes from animal bacteria are transferred to bacteria that are pathogenic to humans [5]. Bacterial resistance is observed when antibiotics are used abundantly, and the bacteria undergo a strong selection pressure [6]. In modern poultry farms, antibiotics are often used for therapeutic, preventive, or growth-stimulating purposes, creating a favorable environment for the development of antibiotic resistance [7,8]. *Escherichia coli* is a bacterium found in the microflora of poultry and is one of the most frequently encountered bacteria in poultry farming, wherein it causes economic losses due to colibacillosis [9]. Among the *E. coli* strains that cause disease in poultry, zoonotic strains are known to be responsible for infections in humans, including strains qualified as extraintestinal pathogenic *E. coli* [10,11]. Salmonellosis also leads to health constraints in poultry farms, resulting in start-up mortality and a drop in laying [12]. In addition to the economic losses in poultry production, *Salmonella* contamination in food is considered to be an issue in international trade (trade barrier) and, above all, a major public health issue (food poisoning) [13].

Thus, given the health risks linked to the presence of *E. coli* and *Salmonella* in both poultry production and human health, as well as the antimicrobial resistance (AMR) issue posed by these germs in public health, regular monitoring of their resistance to antibiotics in poultry farming is essential [14,15]. However, Côte d’Ivoire, like other French-speaking countries of the West African sub-region, does not have a network for monitoring AMR in animal production [16]. However, data on the antibiotic resistance of *E. coli* and *Salmonella* isolated from poultry farms, especially in Côte d’Ivoire, are limited.

Therefore, this study aims to determine the prevalence of the resistance of *E. coli* and *Salmonella* spp. strains from poultry farms in the districts of Abidjan and Agnibilékrou (Côte d’Ivoire) to commonly used antibiotics in veterinary medicine.

## Materials and Methods

### Ethical approval and informed consent

Formal ethical approval was not required for this study, but appropriate poultry farm owner consents were obtained in verbal form before sample collection.

### Study period, location and sample collection

A cross-sectional study was conducted from August to October 2014 on 124 private poultry farms in Abidjan (n=78) and Agnibilékrou (n=46). A total of 231 fecal samples were randomly collected from intensive poultry farms based on the willingness of the farm owners to participate in the study and accessibility of the farms. When a farm consisted of one or two poultry buildings, only one sample was collected. When the farm had more than two poultry buildings, two samples were collected in two different poultry building. A sample consisted of a pool of five fresh feces obtained from the different parts of the poultry buildings. Each farm was visited once, and the samples were collected using tongue depressors and packed in sterile bags. They were immediately stored in coolers containing ice packs and transferred to the storage site and frozen at −20°C before being transported to the laboratory at the EISMV of Dakar (Senegal) without breaking the cold chain where they were stored at the same freezing temperature until the analyses were performed.

### Laboratory analysis

#### Isolation and identification of E. coli and Salmonella

Necessary laboratory equipment and required media were used to culture the target enterobacteriaceae. The isolation of *E. coli* was performed using the method previously described by Vounba *et al*. [17] and identified by classical gallery tests and API 20 E (bioMerieux). *Salmonella* isolation and identification were performed using the method described by Bada-Alambedji *et al*. [18]. The isolates tested positive for *E. coli* and *Salmonella* were sub-cultured onto nutritive agar for antimicrobial susceptibility testing.

### Antimicrobial susceptibility testing

All isolated strains were tested against 14 antibiotics commonly used in veterinary medicine belonging to eight different antibiotic classes: aminoglycosides (streptomycin and Gentamicin), penicillins (amoxicillin and ampicillin), cephalosporins (cefuroxime and ceftriaxone), quinolones (flumequine), furans (nitrofuran), polymyxins (colistin), phenicols (chloramphenicol), tetracyclines (doxycycline and tetracycline), and sulfonamides (sulfonamide and sulfamethoxazole + trimethoprim). A disk-diffusion method was performed and interpreted in accordance with the recommendations of the Antibiogram Committee of the French Society of Microbiology (CA-SFM/EUCAST) [19]. Isolates were categorized as susceptible or non-susceptible to each antimicrobial. An isolate was considered susceptible, if it was sensitive to all of the antibiotics tested and non-susceptible if it was resistant or intermediate to one or more antibiotics.

### Statistical analysis

Data were entered into Microsoft Excel, and the prevalence of antibiotic resistance among the different groups was calculated by dividing the number of resistant isolates in the group by the number of isolates tested. Chi-squared tests were used for statistical analysis of the difference in resistance between the two districts. p<0.05 was considered statistically significant.

## Results

### The number of strains isolated

Out of the 231 samples analyzed, 212 *E. coli* and 36 *Salmonella* strains were isolated. In Abidjan, 139 samples generated 101 *E. coli*, and 23 *Salmonella* strains, whereas in Agnibilékrou, 92 samples generated 111 *E. coli* and 13 *Salmonella* strains.

### Antibiotic resistance of E. coli strains

#### Resistance to beta-lactam antibiotics

Among the classes of antibiotics, resistance was more observed in penicillin than in cephalosporin (Table-1). In strains isolated from poultry farms, a higher resistance was observed in ampicillin (49.53%) and amoxicillin (25.94%) compared with cefuroxime (4.25%) and ceftriaxone (2.36%) (Table-1).

**Table 1:** Resistance of *Escherichia coli* strains to different antibiotics tested.

A-Resistance of *E.coli* strains to Beta-lactam antibiotics								

Class/Antibiotics	Penicillins				Cephalosporins			
	
	Amoxicillin		Ampicillin		Cefuroxime		Ceftriaxone	
			
	Resistance	p-value	Resistance	p-value	Resistance	p-value	Resistance	p-value
ABIDJAN (n=101)	27(26.73%)	0.8025	52(51.49%)	0.5867	6(5.94%)	0.2428	3(2.97%)	0.5755
AGNIBILEKROU (n=111)	28(25.23%)		53(47.75%)		3(2.70%)		2(1.80%)	
Total (n=212)	55(25.94%)		105(49.53%)		9(4.25%)		5(2.36%)	

**B-Resistance of *E.coli* strains to Aminoglycosides antibiotics**								

	**Streptomycin**				**Gentamicin**			
	
	**Resistance**	**p-value**			**Resistance**	**p-value**		

ABIDJAN (n=101)	71(70.30%)	0.6657			8(7.92%)	0.0358		
AGNIBILEKROU (n=111)	81(72.97%)				2(1.80%)			
Total (n=212)	152(71.70%)				10(4.72%)			

**C-Resistance of *E.coli* strains to Tetracyclines and Sulfonamides**								

	**Doxycycline**		**Tetracycline**		**Sulfonamide**		**Trimethoprim-Sulfamethoxazole**	
			
	**Resistance**	**p-value**	**Resistance**	**p-value**	**Resistance**	**p-value**	**Resistance**	**p-value**

ABIDJAN (n=101)	99(98.02%)	0.9240	98(97.03%)	0.9065	87(86.14%)	0.6324	85(84.16%)	0.1665
AGNIBILEKROU (n=111)	109(98.20%)		108(97.30%)		93(83.78%)		85(76.58%)	
Total (n=212)	208(98.11%)		206(97.17%)		180(84.91%)		170(80.19%)	

**D-Resistance to other classes of antibiotics**								

	**Flumequine**		**Chloramphenicol**		**Colistin**		**Nitrofuran**	
			
	**Resistance**	**p-value**	**Resistance**	**p-value**	**Resistance**	**p-value**	**Resistance**	**p-value**

ABIDJAN (n=101)	58(57.43%)	0.7172	21(20.79%)	0.7601	30(29.70%)	0.3003	0(0.00%)	0.0961
AGNIBILEKROU (n=111)	61(54.95%)		25(22.52%)		26(23.42%)		3(2.70%)	
Total (n=212)	119(56.13%)		46(21.70%)		56(26.42%)		3(1.42%)	

#### Resistance to aminoglycosides

A higher resistance was observed in streptomycin (71.70%) compare with that in gentamicin (4.72%) (Section 1-B of Table-1). Moreover, resistance to gentamicin was significantly higher in Abidjan than in Agnibilékrou (p=0.0358).

#### Resistance to tetracyclines and sulfonamides

Higher resistance rates were observed in tetracyclines and sulfonamides, which ranged from 80.9% to 84.91%, for the combination treatment of trimethoprim-sulfamethoxazole and sulfonamide, respectively, and from 97.17% to 98.11% for tetracycline and doxycycline, respectively (Section 1-C of Table-1).

#### Resistance to other classes of antibiotics

Resistance rates observed for other classes of antibiotics such as quinolone, phenicol, polymyxin, and furans were 56.13%, 26.42%, 21.70%, and 1.42% for flumequine, colistin, chloramphenicol, and nitrofuran, respectively (Section 1-D of Table-1)

### Antibiotic resistance of Salmonella strains

#### Resistance to beta-lactam antibiotics

Resistance of *Salmonella* strains isolated from poultry in Abidjan and Agnibilékrou indicated a high resistance for penicillin antibiotics than that for cephalosporin antibiotics, similar to the resistance observed for *E. coli* (Table-2). Resistance observed for ampicillin (44.44%) and amoxicillin (33.33%) was high compared with that for cefuroxime (5.56%) and ceftriaxone (00.00%), as presented in Section 2-A of Table-2.

**Table 2:** Resistance of *Salmonella* spp. strains to different antibiotics tested.

A-Resistance of *Salmonella* spp. strains to beta-lactam antibiotics								

Classes/ Antibiotics	Penicillins				Cephalosporins			
	
	Amoxicillin		Ampicillin		Cefuroxime		Ceftriaxone	
			
	Resistance	p-value	Resistance	p-value	Resistance	p-value	Resistance	p-value
ABIDJAN (n=23)	11(47.83%)	0.5870	7(30.43%)	0.6236	2(8.70%)	0.2739	0(0.00%)	-
AGNIBILEKROU (n=13)	5(38.46%)		5(38.46%)		0(0.00%)		0(0.00%)	
Total (n=36)	16(44.44%)		12(33.33%)		2(5.56%)		0(0.00%)	

**B-Resistance of *Salmonella* spp. strains to aminoglycosides**								

	**Streptomycin**				**Gentamicin**			
	
	**Resistance**	**p-value**			**Resistance**	**p-value**		

ABIDJAN (n=23)	10(43.48%)	0.1371			9(39.13%)	0.1958		
AGNIBILEKROU (n=13)	9(69.23%)				8(61.54%)			
Total (n=36)	19(52.78%)				17(47.22%)			

**C-Resistance of *Salmonella* spp. strains to Tetracyclines and Sulfonamides**								

	**Doxycycline**		**Tetracycline**		**Sulfonamide**		**Trimethoprim-Sulfamethoxazole**	
			
	**Resistance**	**p-value**	**Resistance**	**p-value**	**Resistance**	**p-value**	**Resistance**	**p-value**

ABIDJAN (n=23)	23(100.0%)	0.0529	21(91.30%)	0.0230	21(91.30%)	0.2307	13(56.52%)	0.0162
AGNIBILEKROU (n=13)	11(84.62%)		10(76.92%)		10(76.92%)		2(15.38%)	
Total (n=36)	34(94.44%)		31(86.11%)		31(86.11%)		15(41.67%)	

**D-Resistance to other classes of antibiotics**								

	**Flumequine**		**Chloramphenicol**		**Colistin**		**Nitrofuran**	
			
	**Resistance**	**p-value**	**Resistance**	**p-value**	**Resistance**	**p-value**	**Resistance**	**p-value**

ABIDJAN (n=23)	16(69.57%)	0.6236	0(0.00%)	0.1773	0(0.00%)	0.1773	0(0.00%)	-
AGNIBILE-KROU (n=13)	8(61.54%)		1(7.69%)		1(7.69%)		0(0.00%)	
Total (n=36)	24(66.67%)		1(2.78%)		1(2.78%)		0(0.00%)	

#### Resistance to aminoglycosides

Resistance observed for streptomycin (52.78%) was high compared with that observed for gentamicin (47.22%), as reported in Section B of Table-2.

#### Resistance to tetracyclines and sulfonamides

As for *E. coli*, the highest resistance rates were observed in these classes, with them ranging from 41.67% for the trimethoprim–sulfamethoxazole combination to 86.11% and 94.44% for sulfonamide, tetracycline, and doxycycline, respectively (Section C of Table-2).

#### Resistance to other classes of antibiotics

Resistance observed for other classes of antibiotics such as quinolones, phenicols, polymyxins, and furans was 66.67, 2.78%, and 0.00%, respectively, for flumequine, colistin, chloramphenicol, and nitrofuran antibiotics, as presented in Section D of Table-2.

### Antibiotic resistance pattern of Salmonella serovars

Six different *Salmonella* serovars were identified among the *Salmonella* strains isolated (Table-3). The most prevalent serovars were *Salmonella* Kentucky and *Salmonella* Sandiego. Other serovars were *Salmonella* Agama, *Salmonella* Djugu, *Salmonella* Poona, and *Salmonella* Mbandaka. In all serovars, except *Salmonella* Agama, resistance to at least one antibiotic was 100%. Multi-resistance likely occurred in *Salmonella* Kentucky and *Salmonella* Poona serovars with 93.33% and 66.67% of the strains, respectively, resistant to more than five antibiotics simultaneously.

**Table 3:** Antibiotic resistance profile of *Salmonella* serovars.

Serovars	Number of strains	GMI	FTN	SXT	UBN	SUL	TET	DOX	SMN	CXM	AMP	CST	CRO	CHL	AMX	No resistance	Resistance to 01-05 antibiotics	Resistance to more than 05 antibiotics
S. Agama	3	0	0	0	0	0	0	2	0	1	0	0	0	0	1	1	2 (66.67%)	0
S. Djugu	3	0	0	0	1	2	2	3	0	0	0	0	0	0	2	0	3 (100%)	0
S. Kentucky	15	14	0	2	14	15	15	15	14	0	11	1	0	0	12	0	1 (6.67%)	14 (93.33%)
S. Mbandaka	2	0	0	0	0	1	1	1	2	0	0	0	0	0	0	0	2 (100%)	0
S. Poona	3	2	0	3	3	3	3	3	1	0	1	0	0	1	1	0	1 (33.33%)	2 (66.67%)
S. Sandiego	10	1	0	10	6	10	10	10	2	1	0	0	0	0	0	0	8 (80%)	2 (20%)
Total	36	17	0	15	24	31	31	34	19	2	12	1	0	1	16	1	17	18

GMI=Gentamicin, FTN=Nitrofuran, SXT=Sulfamethoxazole-Trimethoprim, UBN=Flumequine, SUL=Sulfonamides, TET=Tetracycline, DOX=Doxycycline, SMN=Streptomycin, CXM=Cefuroxime, AMP=Ampicillin, CST=Colistin, CRO=Ceftriaxone, CHL=Chloramphenicol, AMX=Amoxicillin

#### Comparison of resistance of E. coli and Salmonella strains

**Figure-1:**
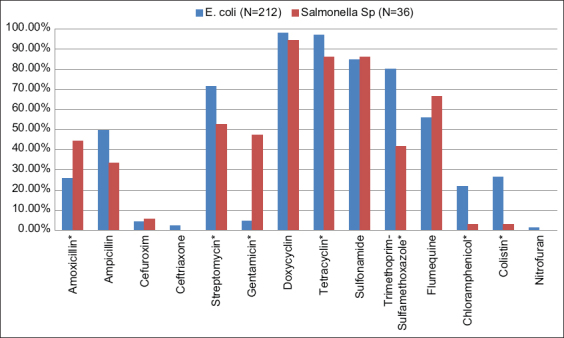
Comparison of resistance of *Escherichia coli* and *Salmonella* spp. strains to antibiotics tested (*mean difference is significant).

Globally, except for amoxicillin, cefuroxime, gentamicin, sulfonamide, and flumequine, the resistance of *E. coli* strains was higher than that of *Salmonella* strains. The difference in resistance was significant (p<0.05) for antibiotics such as streptomycin, tetracycline, trimethoprim-sulfamethoxazole, chloramphenicol, and colistin (Figure-1).

## Discussion

*E. coli* is a Gram-negative, facultative anaerobe bacterium of the Enterobacteriaceae family. Since *E. coli* is ubiquitous in the gastrointestinal tract of warm-blooded animals, it has been extensively used to monitor AMR in food animals (including poultry) [14,15,20]. Moreover, some *E. coli* strains hosted by poultry are a potential source of AMR genes that could be transmitted to humans [21]. Part of this research work focused on the antibiotic resistance of *E. coli* from poultry farms and reported different levels of resistance. Resistance observed in beta-lactams was high against penicillins and very low against cephalosporins. Similar resistance rates were reported in France by the Résapath network in 2018 for *E. coli* from poultry which were 25% and 29%, respectively, for ampicillin and amoxicillin and less than 5% for cephalosporin [22]. The low resistance rates observed for cephalosporins in this study can be due to the fact that in Côte d’Ivoire, third- and fourth-generation cephalosporin antibiotics (C3G and C4G) are not common in veterinary medicine, especially in food-producing animals such as poultry. The selection of resistance to third-generation cephalosporins with the production of BLSE is certainly largely attributable to the use of C3G/C4G in veterinary medicine, despite the co-selection by other antibiotics (tetracycline or sulfonamide drugs in animals) [23].

According to the results, *E. coli* resistance was very high for tetracycline and sulfonamide antibiotics, such as doxycycline (98.11%), tetracycline (97.17%), sulfonamide (84.91%), and trimethoprim-sulfamethoxazole (80.19%). Similar high resistance rates were also reported for these antibiotics in other studies conducted in Senegal, wherein *E. coli* isolated from healthy chicken farms displayed a high prevalence of AMR to trimethoprim-sulfamethoxazole (76.7%), sulfisoxazole (80.8), and tetracycline (92.2%) [24]. These high resistance rates could be explained by the fact that these antibiotics are the most commonly purchased in such countries and the most used in poultry farms. Indeed, tetracycline and sulfonamide antibiotics are the most used antibiotics in modern poultry farms in many sub-Saharan African countries, with sulfonamide antibiotics being used as an anti-parasite treatment for coccidiosis [25]. However, the antibiotic resistance in the present study was higher than that reported in Canada from small poultry flocks [26], wherein resistance to streptomycin (71.70%) was higher than the resistance found in Senegal.

In this study, resistance to gentamicin (4.72%) was similar to the resistance reported in France, wherein *E. coli* from poultry remains predominantly sensitive to aminoglycosides, such as gentamicin, for which the proportions of sensitivity are ≥97% [22].

Moderate resistance to some antibiotics, such as flumequine (56.13%), colistin (26.42%), chloramphenicol (21.70%), and nitrofuran (1.42%), was observed. Moreover, resistance to quinolones (flumequine) and colistin antibiotics are higher in the present study than those reported in other studies [27]. Such resistance should be investigated further as these antibiotics, especially colistin, are important in human health.

Chloramphenicol and nitrofuran are not authorized for use in poultry. Therefore, the resistances observed for these antibiotics may be due to a co-selection or illegal use of these antibiotics sold in illegal vet drug markets that are not secure in sub-Saharan African countries [28]. The co-selection hypothesis is supported by the findings of Bischoff *et al*. [29], who suggested that a mechanism for co-selection and maintenance of chloramphenicol resistance in pathogenic *E. coli* exists in the absence of direct selection pressure from phenicol use. According to these authors who observed co-resistance to sulfamethoxazole, tetracycline, and kanamycin among the majority of chloramphenicol-resistant trans-conjugants, the use of any of these antimicrobials can result in the selection of bacteria resistant to not only that specific agent, by genetic linkage of resistance genes but also other unrelated antimicrobial agents. Since the resistance to trimethoprim-sulfamethoxazole combination and tetracycline was the highest resistance reported in the present study, the resistance observed for chloramphenicol antibiotics can be easily explained.

The present study also assessed the antibiotic resistance of *Salmonella* from feces, and similar levels of resistance were observed for *E. coli*. Resistance to penicillins was high for beta-lactamins (amoxicillin, 44.44% and ampicillin, 33.33%) and very low for cephalosporins (cefuroxime, 5.56% and ceftriaxone, 0.00%). The combined data from the Résapath network and the *Salmonella* network in France corroborate these very low proportions of *Salmonella* strains of animal or environmental origin resistant to cephalosporins [23].

According to the results, *Salmonella* resistance was also very high for tetracycline (doxycycline [94.44%] and tetracycline [86.11%]) and sulfonamide antibiotics (sulfonamide [86.11%] and trimethoprim-sulfamethoxazole [41.67%]). These resistance rates of *Salmonella* are higher than those reported in a study conducted in Kenya, wherein resistance to co-trimoxazole, tetracycline, and streptomycin was 28%, 11%, and 6%, respectively [30]. However, these results are similar to those reported in other studies in Ethiopia [31] and Ghana [32], wherein the researchers reported resistances of 100% and 82% for tetracycline and 69% and 56% for trimethoprim-sulfamethoxazole.

Resistance to gentamicin (47.22%) and flumequine (66.67%) was higher than the resistance reported by Cui *et al*. [33], who reported resistance of 6.8% and 41.1%, respectively, for gentamicin and first-generation quinolone. The high resistance rates observed in this study should be considered because these antibiotics, in particular quinolones, are clinically important antibiotics in human health [34]. The identified serovars are common in the poultry industry [35]. Apart from the *S*. Sandiego serovar, various serovars had already been identified in carcasses, gizzards, and pieces of poultry meat sold in various markets in the country [36,37]. The *S*. Kentucky serovar, the most isolated in our study (42%) and the most likely to harbor multi-resistant strains, was previously reported and subject of alarm by the national *Salmonella* surveillance systems from France, England, Denmark, and the United States because these surveillance systems identified the emergence of multidrug-resistant isolates of *Salmonella* *enterica* serotype Kentucky with high resistance to antibiotics, with poultry being the main reservoir and vehicle for human infections [38].

The high proportion of resistance observed, with 97% of the 36 isolates harboring resistance to at least one antibiotic, suggests that therapeutic options could be limited in the treatment of salmonellosis in the poultry farms of Côte d’Ivoire, with *Salmonella* spp. being bacteria that are associated with great losses in animal production and with public health concerns because of their role as zoonotic and foodborne pathogens [39].

It has been demonstrated that limiting antimicrobial use reduces AMR in food animals and probably reduces AMR in humans, even if the magnitude of the effect is not yet quantified [40]. Moreover, the use of antibiotics in poultry in Côte d’Ivoire should be regulated to reduce the levels of resistance as were observed in the present study. In Côte d’Ivoire [36,41] or in other sub-region countries (Senegal [18,42] and Burkina Faso [43]), the poultry carcass has been demonstrated to harbor antibiotic-resistant *Salmonella* coming probably from the primary production and the lack of hygiene in slaughterhouses. Therefore, controlling the antibiotic resistance in primary production will protect consumers and public health against resistant bacteria or resistant genes that can be transmitted to humans through the food chain.

## Conclusion

The overall prevalence of antibiotic resistance of *E. coli* and *Salmonella* in poultry farms in Côte d’Ivoire should be investigated further. Efforts are crucial to reduce antibiotic resistance in poultry, including the adoption of guidelines for prudent use of antimicrobial agents in animals intended for food and regulation on the access to antimicrobials. In Côte d’Ivoire, like in other developing countries, the indiscriminate and widespread use of antimicrobials in veterinary practice and the easy access to antimicrobials by farmers who can purchase them without any prescription should be addressed. The resistance to the relatively cheaper and commonly available antimicrobials (tetracycline and sulfonamides) reported here is alarming as these resistances will lead to more expensive therapies and a longer duration of animal sickness resulting in lower production levels in farms. The resistant pattern of *E. coli* and *Salmonella* in poultry to clinically important antibiotics in humans, such as quinolones and penicillins, that are used for treating infections is a concern because chickens could be a source of multidrug-resistant bacteria or bacteria genes in humans. We believe that, based on the evidence reported here, efforts should be concentrated on the control of antibiotic resistance at the farm level in Côte d’Ivoire.

## Authors’ Contributions

MAA, ATA, WO, KA, and RBA conceptualized and designed research. MAA, ATA, WO, KA, RBA, FN, SHZ, AAZ, RCTT, VK, APB, and KD contributed in sample collection and/or samples or data analysis. APB wrote the first manuscript draft. MAA, KA, WO, and RBA edited and revised the final draft of the article. All authors have critically reviewed the manuscript and approved the final version.
